# Processing Interrogative Sentence Mood at the Semantic-Syntactic Interface: An Electrophysiological Research in Chinese, German, and Polish

**DOI:** 10.1371/journal.pone.0013036

**Published:** 2010-09-29

**Authors:** Chung-Shan Kao, Rainer Dietrich, Werner Sommer

**Affiliations:** 1 Département de Psychologie, Université de Fribourg, Fribourg, Switzerland; 2 Institut für deutsche Sprache und Linguistik, Humboldt-Universität zu Berlin, Berlin, Germany; 3 Institut für Psychologie, Humboldt-Universität zu Berlin, Berlin, Germany; L'université Pierre et Marie Curie, France

## Abstract

**Background:**

Languages differ in the marking of the sentence mood of a polar interrogative (yes/no question). For instance, the interrogative mood is marked at the beginning of the surface structure in Polish, whereas the marker appears at the end in Chinese. In order to generate the corresponding sentence frame, the syntactic specification of the interrogative mood is early in Polish and late in Chinese. In this respect, German belongs to an interesting intermediate class. The yes/no question is expressed by a shift of the finite verb from its final position in the underlying structure into the utterance initial position, a move affecting, hence, both the sentence's final and the sentence's initial constituents. The present study aimed to investigate whether during generation of the semantic structure of a polar interrogative, i.e., the processing preceding the grammatical formulation, the interrogative mood is encoded according to its position in the syntactic structure at distinctive time points in Chinese, German, and Polish.

**Methodology/Principal Findings:**

In a two-choice go/nogo experimental design, native speakers of the three languages responded to pictures by pressing buttons and producing utterances in their native language while their brain potentials were recorded. The emergence and latency of lateralized readiness potentials (LRP) in nogo conditions, in which speakers asked a yes/no question, should indicate the time point of processing the interrogative mood. The results revealed that Chinese, German, and Polish native speakers did not differ from each other in the electrophysiological indicator.

**Conclusions/Significance:**

The findings suggest that the semantic encoding of the interrogative mood is temporally consistent across languages despite its disparate syntactic specification. The consistent encoding may be ascribed to economic processing of interrogative moods at various sentential positions of the syntactic structures in languages or, more generally, to the overarching status of sentence mood in the semantic structure.

## Introduction

Speech production involves mental processes of transforming a holistic idea into a serial string of words uttered in succession. According to the prevalent modeling, utterance generation proceeds at several processing levels [1], [2]. At first, speakers select contents for expression aiming to convey their communicative intention. The contents of an utterance are then arranged to create a semantic structure in a propositional form. This semantic representation is further mapped onto linguistic forms. The mapping entails two levels: grammatical formulation, yielding a surface structure of the sentence, and phonological specification, inserting word forms into the syntactic frame. Finally, the phonetic plan of the utterance is articulated by executing motor programs for movements of speech organs.

**Figure 1:**
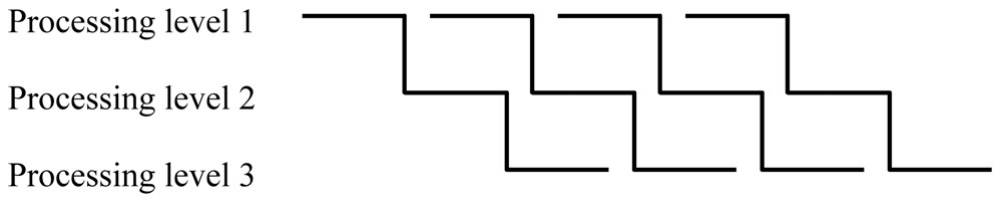
A simplified diagrammatic depiction of incremental speech production [1], [2]. As soon as the encoding of an utterance constituent is finished at one processing level, it triggers its processing at the ensuing level. Meanwhile, the next constituent is processed at the foregoing level. Different constituents are hence encoded parallel at several processing levels, one constituent at each level, respectively.

Researchers assume that a complex utterance is not processed holistically at each processing level. Instead, the production is implemented in an incremental way, particularly from semantic structuring towards articulation, as shown in Figure 1 [1], [3]–[7]. At each processing level the constituents of an utterance are encoded serially. Ideally the encoding order will be in line with the order of mention. Furthermore, as soon as the encoding of a constituent is accomplished at one level, its encoding at the succeeding level is launched. Meanwhile, the next constituent is encoded at the previous level. Thus, different constituents are processed concurrently and in parallel at several levels – one constituent at each level.

There is ample evidence in favor of such serial-parallel utterance production. Lexical information of the utterance constituents to be encoded at a specific processing level – be it semantic, syntactic, or phonological– is activated sequentially in the order of mention [8]–[10]. Studies of eye movements during picture viewing indicate that the fixations of objects, which reflect lexical selection and phonological encoding of an utterance constituent, anticipate the exact order of mention of these objects in the description [11]–[13]. Finally, the constituents of utterances are encoded in sequence at each processing level. For instance, the sentence frame of an utterance is incrementally constructed at the level of grammatical encoding [14].

The present study addressed the issue of when the sentence mood of an utterance is processed at the semantic-syntactic interface. Levelt postulates that the semantic structure of an utterance is marked for sentence mood [1]. For instance, the semantic processing of a question entails generating a global structure in form of a hierarchical constellation of local function/argument structures and coding the global structure with an interrogative marker. The mood marker represents a function that modifies the whole proposition of an utterance and therefore takes a high position in the semantic structure. We posit that during semantic processing of an utterance the sentence mood is encoded either before or after the entire proposition. However, the encoding order of the proposition and the mood of a specific sentence type should not vary from utterance to utterance. Rather, in a given language the two sentential parts are encoded always in one and the same of the two possible orders, for example, proposition before mood or mood before proposition.

A plausible determinant of the fixed encoding order in a given language arises from the incremental processing of speech production. The processing of an utterance at the semantic-syntactic interface will be maximally incremental when sentence constituents are encoded semantically and syntactically in the same sequence. Since the syntactic encoding order of the proposition and the sentence mood of an utterance is contingent on the grammar of a language, their semantic encoding ought to be implemented in the same order as well. For instance, a Polish declarative (1) of depicting a telephone behind a lattice differs from a typical polar interrogative (yes/no question) (2), which expresses one and the same proposition, in the question particle at the beginning of the interrogative:

1. *Telefon jest z tyłu*.
  telephone is behind (the lattice)
  ‘The telephone is behind (the lattice).’
2. *Czy telefon jest z tyłu?*
  Q-PART. telephone is behind (the lattice)
  ‘Is the telephone behind (the lattice)?’
  In order to incrementally construct the sentence frame of a Polish polar interrogative, the interrogative mood should be syntactically encoded prior to the proposition. If the two sentential parts are encoded semantically in the same sequence, the interrogative mood will be encoded before the proposition during generation of the semantic structure in Polish. In Chinese, by contrast, the interrogative mood is marked by a particle at the end of a typical yes/no question (4):

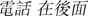

  telephone behind (the lattice)
  ‘The telephone is behind (the lattice)’

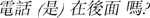
>
  telephone (be) behind (the lattice) Q-PART.
  ‘Is the telephone behind (the lattice)?’
  On the incremental tenet of utterance production, the sentence mood of a Chinese polar interrogative will hence be semantically encoded after the proposition.
  The interrogative mood of a typical yes/no question in German (6) is marked, in comparison to its declarative counterpart, by coding the finite verb in the sentence initial position:
5. *Das Telefon ist hinten*.
  the telephone is behind (the lattice)
  ‘The telephone is behind (the lattice)?’
6. *Ist das Telefon hinten?*
  is the telephone behind (the lattice)
  ‘Is the telephone behind (the lattice)?’

Theoretically there are two possible encoding orders, which contradict each other. In a strictly incremental feed-forward system, the interrogative mood will be encoded before the proposition during semantic structuring of a polar interrogative in German as in Polish. On the other hand, in the framework of generative grammar specific theoretical constraints lead to the conjecture that all surface structures in a given language are derived from one basic underlying frame [15]. Hence the underlying frame of a certain language has a fixed constituent order in terms of the finite verb and its two arguments, subject and object. A number of German grammarians claim that the verb is originally located at the sentence end of the German underlying frame [16]–[20]. In order to generate the surface structure of a polar interrogative the finite verb will then be moved to the sentence initial position. According to this premise, the semantic encoding order of German polar interrogative will accord with the underlying frame, which is the first syntactic structure generated in the grammatical encoding. That is to say, as in Chinese, the German interrogative sentence mood, coded by the finite verb, will be processed after the propositional content.

In contrast to the language-specific contention, it is possible that the encoding of the sentence mood is not implemented incrementally by virtue of the special mood marker function, which takes the complete proposition of an utterance as its argument in the semantic structure. Instead, it may be the unique status of the proposition-modifying function of the mood marker that determines the semantic encoding order of the two sentential parts. In this regard the proposition and the sentence mood of an utterance are supposed to be encoded in a predetermined sequence across all sentence types and all languages. For instance, during the semantic structuring of a polar interrogative, the sentence mood may be encoded consistently either before or after the proposition in all languages. That is, despite the aforementioned disparity of the syntactic encoding order in Chinese, German, and Polish, the semantic encoding order of the two sentential parts may not differ between languages.

In the present study native speakers of Chinese, German, and Polish performed a two-choice go/nogo task while the electroencephalogram (EEG) was registered to clarify the relative temporal order of encoding sentence mood and propositional content of a polar interrogative. To investigate the order of accessing lexical information (semantic, syntactic, and phonological), researchers of speech production have recently employed electrophysiological measures such as the lateralised readiness potential, abbreviated as LRP [21]–[28]. The LRP is a brain potential derived from the recorded EEG and related to a motor event [29]–[31]. If one prepares a voluntary hand movement, a negative potential develops over the scalp [32]. One of the generators of this readiness potential (RP) is the primary motor cortex [33]. In the initial phase of the preparation the RP emerges symmetrically and then lateralizes to the hemisphere contralateral to the moving hand [34].

An LRP emerges from movement preparation, independent of whether the prepared movement is actually executed (go) or withheld (nogo). This property gives rise to the exploitation of the LRP as indicator of processing order in two-choice go/nogo paradigms [35], [36]. Van Turennout et al., for instance, examined the time course of retrieving syntactic and phonological information during lexical access [28]. Line drawings of objects or animals were presented one after another on a screen. Dutch-speaking participants responded to each picture by pressing either a left button with the left hand (go), a right button with the right hand (go), or none of them (nogo). In go trials the choice of the responding hand (left or right) was connected with the grammatical gender of the picture name. In Dutch there are two nominal genders: common gender *de* (e.g. *de beer* ‘the bear’ and *de schoen* ‘the shoe’) and neuter gender *het* (e.g. *het boek* ‘the book’ and *het schaap* ‘the sheep’). The participants were instructed to press one key in response to the common gender and the other key in response to the neuter gender. The decision of response execution (go or nogo) was connected to the initial phoneme of the picture name. For example, participants had to execute a hand movement if the object name began with/b/as in *beer* and *boek*. If the initial phoneme was/s/as in *schoen* and *shaap*, no response was to be made.

Thus, in the experimental design two motor processes of the button pressing task, the hand choice (left or right) and the execution decision (go or nogo), were connected with two kinds of lexical information. The key observation was the presence or absence of an LRP in the nogo trials, which was predicted to hinge on the temporal relation of the hand choice and the nogo decision. If the phoneme retrieval of a noun was contingent on the activation of its gender, the nogo decision (connected with the phoneme retrieval) could be made only after a hand choice (according to noun gender), thereby yielding a nogo-LRP. That is, the emergence of a nogo-LRP would indicate that the syntactic information of a word is retrieved prior to phonological information. Van Turennout et al. found that an LRP emerged in the nogo trials, lending credence to their prediction.

## Main Experiment

The present investigation used the LRP in a two-choice go/nogo design to examine the encoding order of two sentential parts of a polar interrogative – interrogative mood and proposition – during semantic structuring of an utterance in Chinese, German, or Polish. In the experimental design we attempted to connect the hand choice with the encoding of a propositional content and the nogo decision with the encoding of the interrogative mood. The emergence of a nogo-LRP should then disclose the encoding order of the two sentential parts when speakers generate the semantic structure of a polar interrogative in their native language.

**Figure 2:**
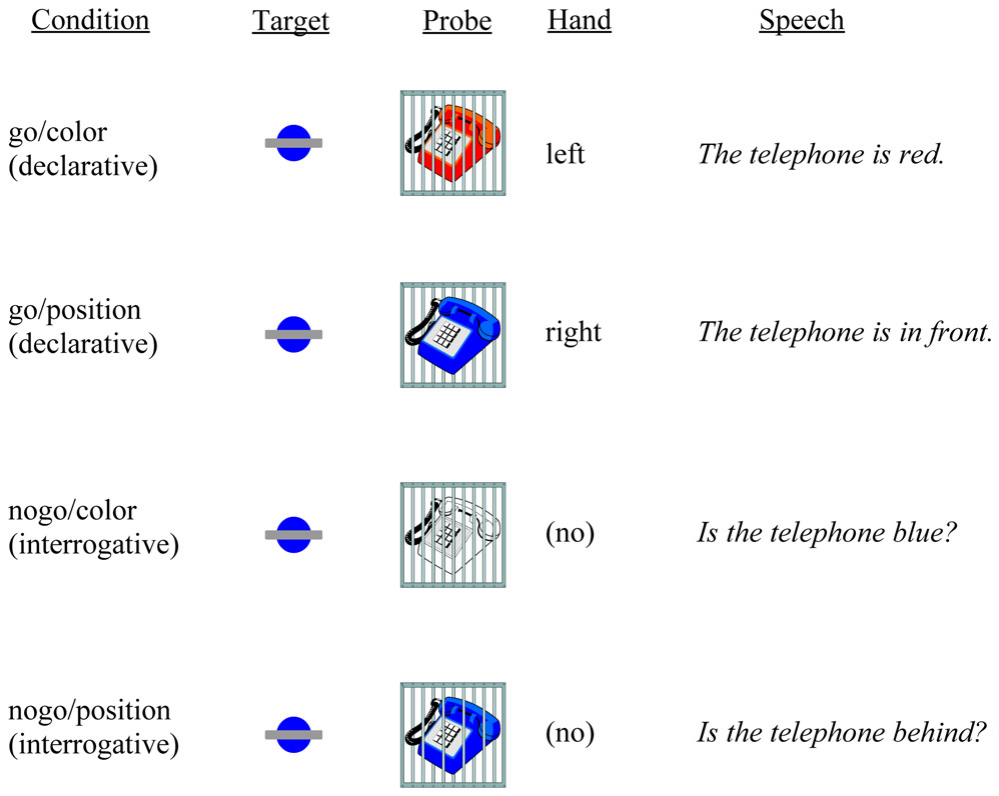
Examples of the four experimental conditions in a two-choice go/nogo design. In each trial two pictures were presented one after the other on screen. The first picture, the target, consisted of a colored circle in front of or behind a gray, horizontal oblong. The second picture, the probe, comprised a colored object in front of or behind a gray lattice of iron bars. In the go/color condition the probe color deviated determinably from the target color. In the go/position condition it was the probe position determinably deviating from the target position. In the nogo/color condition the deviated probe color was indeterminable. In the nogo/position condition the probe object was stuck between the iron bars, giving rise to an indeterminable position. Participants were instructed to respond to each condition accordingly with button pressing and speaking.

Native speakers of Chinese, German, and Polish responded to picture stimuli by pressing a button (or refraining from doing so) and uttering a sentence. In each trial two pictures were presented one after the other on the screen. The first picture, the target, consisted of a colored circle before or behind a gray, horizontal oblong (e.g. a blue circle behind the oblong). The second picture, the probe, showed a colored object before or behind a gray lattice of bars (e.g. a red telephone behind or a blue telephone before the lattice; see Figure 2). The two target attributes, color and position, represented the color and position to be matched in the probe stimulus. One of the attributes of the probe, either the color or the position, deviated from the target attribute. In go trials the deviant attribute of the probe was clearly determinable. That is, color or position deviated from the target stimulus. Participants had to merely disconfirm the deviant attribute by pressing the corresponding button and by verbally describing the deviating attribute in a declarative sentence (e.g. ‘The telephone is red’ or ‘The telephone is behind [the lattice]’). In nogo trials one attribute of the probe was indeterminable. The object was either colorless (white) or appeared to be stuck between the bars. In this case participants were to refrain from pressing any button and to ask whether the indeterminable attribute corresponded to that of the target (e.g. ‘Is the telephone blue?’ or ‘Is the telephone behind [the lattice]?’).

In the experimental design the hand choice (left, right) was coupled with a propositional content (color, position) of the utterance and the execution decision (go, nogo) was coupled with the sentence mood (declarative, interrogative). Note that the participants asked yes/no questions exclusively in the nogo conditions, whereas they formulated declarative sentences in the go conditions. The emergence of a nogo-LRP indicated the processing order of the propositional content and the interrogative mood. If the two sentential constituents are encoded at the semantic level in the same order across languages, the results of Chinese, German, and Polish native speakers ought to be consistent: A nogo-LRP is either elicited or absent in all speakers. On the other hand, if the two sentential constituents are ordered according to their syntactic encoding order in each language, contrasting results are predicted in the three speaker groups. If the interrogative mood is encoded before the proposition of a polar interrogative in Polish, the nogo decision (connected with mood) would occur before any hand choice (connected with propositional content). Thus, no (nogo-)LRP was expected in Polish. If Chinese speakers, on the contrary, encode mood after content when conceptualizing a polar interrogative, they would decide not to execute the reaction which has been chosen and prepared on the basis of the earlier content encoding, leading to the development of a lateralized readiness potential. Therefore, a nogo-LRP was predicted for the Chinese speakers. A nogo-LRP may or may not emerge in German, depending on the assumption about the underlying syntactic frame.

In addition to the nogo-LRPs, the latencies of the hand choice and the nogo decision, were compared between the three speaker groups. In the experimental design the hand choice was connected with the encoding of the propositional content of utterances. Hence hand choice latencies would reveal whether the propositional content was encoded at a comparable time point in the three languages. In order to obtain estimates of hand choice latencies the reaction times (i.e. the interval between the stimulus and the response) was bisected into two parts: the interval from the stimulus to the LRP onset (the S-LRP-interval) and the interval from the LRP onset to the response (the LRP-R-interval). If there were significant differences of reaction times between groups or conditions, these effects should also be manifest in the S-LRP-interval and/or in the LRP-R-interval [37]. In a study of specifying the functional locus of the LRP, Masaki et al. found that the LRP begins after response (hand) choice but prior to motor programming [38]. Therefore, in the present experiment the LRP onset would reflect the time point at which the response hand has been chosen.

In the present experimental design the nogo decision was connected to the encoding of the interrogative mood. The latency of the nogo decision would reveal whether the interrogative mood was encoded at a comparable time point in the three languages. In order to estimate the latency of the nogo decision, we subtracted the nogo- from the corresponding go-LRP for each participant of the three speaker groups. An LRP begins when the response hand is chosen and the motor cortex prepares the hand movement. Thus, the go- and the nogo-LRP usually emerge at the same time. That is, if the two stimulus-locked curves of these LRPs are graphically overlaid, they deflect synchronously from the baseline. The two deflections start to diverge once the nogo decision is made. Whereas the go-LRP continues to increase, the nogo-LRP abates and returns to the baseline by virtue of the withdrawal decision. As a consequence, if the nogo-LRP is subtracted from the corresponding go-LRP, the onset latency of the difference curve would manifest the latency demanded by the nogo decision. In short, the comparison of the nogo decision latencies between the three speaker groups was conducted by creating the difference curve for each group and measuring the onset latencies of the difference curves.

### Method

#### Participants

Twenty-three Chinese (11 female, aged 21 to 36), 20 German (11 female, aged 20 to 39), and 19 Polish (10 female, aged 19 to 27) native speakers participated in the experiment. All participants had been born and raised in the environment of their native language at least until graduation from secondary school. All of them were right-handed and had normal or corrected-to-normal visual acuity. The data of four Chinese participants (one female) were discarded from the results. Three of them had committed errors in more than 25% of the trials in one of the conditions. The fourth one generated so little LRP that the maximum of the response-locked LRP, which should have manifested a considerable amplitude, did not exceed 0.03 µV.

Participants were either paid or received credits required for the psychology curriculum. They gave their consent for participating in the experiment by reading and signing a consent form. The research project was evaluated and supported by the German Research Society and approved by the Ethik-Kommission des Instituts für Psychologie der Humboldt-Universität zu Berlin (Ethics Committee of the Department of Psychology at the Humboldt University of Berlin).

#### Materials

All pictures had the approximate size of 3.5 cm×3.5 cm. The target pictures without frame consisted of a colored circle before or behind a horizontal, gray oblong. There were eight target variants: 4 colors (blue, yellow, green, red) ×2 positions (in the front or back). The probe pictures showed a colored object before or behind a gray lattice. The stimulus set of probes encompassed 30 different objects. Eight variants were created for each object in go trials: 4 colors (blue, yellow, green, red) ×2 positions (in the front or back). In nogo trials intended to induce a polar interrogative regarding color, the object was white and either before or behind the lattice. In nogo trials regarding position, the object had one of four colors (blue, yellow, green, red) and appeared to be stuck between the bars of the lattice. The lattice comprised nine bars within a frame. The stuck object was in front of the uneven, yet in back of the even bars.

#### Design

The three experimental factors were Attribute (color, position), Execution Decision (go, nogo), and Language (Chinese, German, Polish). Each participant performed the two-choice go/nogo task under four experimental conditions: go/color, go/position, nogo/color, and nogo/position. The 480 trials for each participant were divided evenly into 20 blocks. Each of the two go conditions (go/color, go/position) encompassed eight trials and each of the two nogo conditions (nogo/color, nogo/position) two trials in a block. The high proportion of go-trials was chosen in order to encourage response preparation also in nogo conditions [39]. Finally, four additional go-trials in each block served as catch trials which comprised two trials in each of the two go conditions.

As opposed to the previous EEG studies of naming in which button presses had been contingent on retrieval of lexical information, participants in the present two-choice go/nogo experiment might separately carry out button presses, followed by speaking. Therefore, catch trials were introduced to enhance the intended connections of linguistic processes with response choice and execution. In catch trials the duration of stimulus presentation was reduced and participants were required to speak immediately without pressing a button. Thus participants had to prepare linguistic and motor responses in parallel upon stimulus presentation instead of processing them successively.

Six stimulus lists were created from the prepared materials. The lists had the same trial sequence in terms of conditions and differed only in the variants of targets and of probes. Cooccurrence of color and position features of the target and probe pictures were equally probable for all conditions of a list. The presentation frequency of the thirty objects in all attribute combinations were balanced across the six lists. The trial sequence used in each list was randomized with the following constraints: No variant of attributes or objects was repeated within three consecutive trials and in no block were nogo- and catch-trials presented in the first two trials or in succession. Each list incorporated seven practice blocks. The first two blocks contained only go trials. The third practice block consisted in equal parts of go and catch trials. All trials in the fourth practice block were nogo trials. The remaining three practice blocks simulated experimental ones.

#### Procedure

Participants were tested individually in a sound-attenuated and dimly lit chamber. They sat circa 80 cm in front of a computer screen; at its center stimuli were presented against white background. At first, in a familiarization phase participants named all 30 objects one after the other three times. This was followed by six practice blocks, after which the 20 experimental blocks were carried out, separated by self-terminated breaks. The assignment of attributes (color, position) to hands (left, right) was reversed in the second half of the session. Hence participants performed a practice block with the reversed assignment before the second half of the session. All instructions were given both orally and written in the native language of the participants.

Each trial started with a fixation cross shown for 500 ms. The target picture replaced the fixation and stayed on for 1000 ms, followed by another fixation cross for 500 ms. The succeeding probe picture was presented for 2000 ms if the participant pressed no button within this period. Otherwise, a button press terminated the probe presentation. In catch trials the probe duration was reduced either to 400 ms or to 800 ms when no button was pressed. After the probe a caricature of a manikin with an empty speech bubble appeared for 2500 ms. The interval between trials, during which participants saw a white screen without stimulus, lasted for 2000 ms.

The participants’ task consisted of button pressing and speaking. According to the outcome of the comparison of the probe with the target, the participants had to press one of the buttons as soon as possible in go conditions (including catch trials) but to refrain from doing so in nogo conditions. With respect to speaking participants had to utter a sentence upon the presentation of the caricature. In experimental go trials, the button press triggered the appearance of the caricature. In catch trials, however, participants were to articulate immediately upon the appearance of the caricature, no matter whether they had already pressed a button or not. In nogo trials, the caricature appeared always 2000 ms after the probe onset.

The error rates of catch trials were analyzed to examine their efficacy. An error occurred in catch trials when participants pressed a button albeit the caricature had appeared on screen, suggesting that motor processes were based on preliminary results of sentence production. The analysis revealed that the average error rate was below 0.25% in the color condition and below 0.20% in the position condition. The low error rates indicate that catch trials may have, as intended, contributed to the synchronization of motor and speech production processes.

#### Electrophysiological recordings

The EEG was recorded with 28 Ag/AgCl electrodes mounted in an elastic cap (Easycap GmbH, Germany) at the scalp sites Fp1, Fp2, Fz, F3, F4, F7, F8, FC1, FC2, FC5, FC6, Cz, C3, C4, T7, T8, CP1, CP2, CP5, CP6, Pz, P3, P4, P7, P8, Oz, O1, O2, and A2, according to the revised international 10–20 system [40]. To record the electrooculogram (EOG) four external electrodes were placed at the outer canthi for horizontal and at the infraorbital rims below both eyes for vertical eye movements. The electromyogram (EMG) was recorded by four electrodes affixed to the forearms to measure peripheral response activations of forefingers for button presses [41]. Electrode impedances were kept below 5 kiloohm. All channels were referenced initially to the left mastoid (A1) with an electrode at AFz as ground and rereferenced offline to the average of these channels. A Brainamp AC amplifier (Brain Products GmbH, Germany) digitized the recorded signals at a sampling rate of 250 Hz. The bandpass for EEG and EOG was set from 0.01 Hz to 70 Hz and the bandpass for EMG from 0.01 Hz to 120 Hz.

#### Data analyses

The catch trials were excluded from all analyses. Go or nogo trials were excluded in the following cases: First, participants pressed the incorrect button in go or any button in nogo conditions; in go trials the reaction time (the latency of button press) was shorter than 300 ms or longer than 2000 ms. Second, the participant uttered incomplete or no sentence; the inspection sentences for formulation focused on sentence mood (declarative or interrogative) that had to accord with the experimental condition (go or nogo). Any go trial with an interrogative utterance or nogo trial with a declarative sentence was eliminated from further data analyses. Aberrant object names or attributes and small speaking impediments such as slight deferment, stutter, or repetition were tolerated in that they should have been irrelevant to the mental processes under investigation.

The analysis of the EEG data began with an ocular artefact correction by the procedure of *multiple source eye correction* (MSEC, as implemented in the BESA software, MEGIS Software GmbH, Germany) [42]. The corrected, continuous EEG was partitioned into 2500 ms segments, 1000 ms before and 1500 ms after the stimulus onset (i.e. the probe presentation). The baseline correction was conducted on the 100 ms interval before stimulus onset. Then the 2500 ms segments were trimmed to extract the 1200 ms stimulus- and response-locked segments, respectively: The stimulus-locked segments started 200 ms prior to the stimulus onset and ended 1000 ms thereafter. The response-locked segments, by contrast, were tailored between 1000 ms before and 200 ms after the button press response but retained the original baseline-correction prior to the stimulus.

The LRP was derived for each of the four experimental conditions (go/color, go/position, nogo/color, nogo/position). The first step was to calculate the EEG difference between the two scalp sites above the primary motor cortex, C3 on the left and C4 on the right hemisphere. The calculation was undertaken separately for the left and right hand assignment under each condition. In both cases the potential at the ipsilateral electrode was subtracted from that at the contralateral electrode in every trial. The difference potential was then averaged for each hand assignment. Finally, the averaged difference waves for left- and right-hand responses, respectively, were averaged to eliminate systematic asymmetric potentials unrelated to movement preparation [30], [31].

To assess the presence of an LRP the stimulus-locked waveforms from 300 ms to 700 ms after the probe onset were sectioned into twenty successive time windows of 20 ms. Within each time window a one-tailed *t* test was performed by calculating the averaged amplitude against baseline. The existence of an LRP, especially in nogo conditions, was defined as a negative-going wave form that deviated from the baseline for at least 100 ms. That is, the averaged amplitude deviated from zero (indicated by significant *t* tests) in at least five successive 20-ms time windows.

LRP onset latencies were estimated by identifying the first time point at which the LRP exceeded a predefined threshold [37], [43]–[45], namely 0.5 µV for the stimulus-locked LRP in the go conditions, 0.35 µV for the stimulus-locked LRP involving nogo trials (yielding S-LRP-intervals), and 2 µV for the response-locked LRP (LRP-R-interval). If the comparison of S-LRP-intervals involved both go and nogo conditions, the amplitude criterion of 0.35 µV was employed for estimating onset latencies in both conditions. Instead of providing absolute LRP onset latency, this procedure yields relative timing differences between groups or conditions with which we were concerned. That is, albeit LRP onset was defined as the time point at which the waveform deflected from the baseline (amplitude zero), the exact point of deflection was often vague. Hence relative onset latency differences could be estimated more reliably when a sizeable LRP had developed; for a similar argument see Miller at al. (1998). Furthermore, in order to reduce onset estimate errors by virtue of individual differences or residual noise, the original data were transformed by a jackknife-based method. All *F* values for jackknifed data were corrected [44], [46].

Nogo decision latencies were estimated by subtracting the nogo-LRP from the corresponding go-LRP for each participant, both of which had undergone the jackknife-based transformation. The latency of the nogo decision was defined as the first time point at which the difference curve exceeded 0.5 µV.

### Results

#### Behavioral data

Table 1 shows the means and standard deviations of reaction times and error rates. Analyses of variance (ANOVA) with factor Group (Chinese, German, Polish) indicated a marginal difference of the reaction times between the speaker groups, *F*(2, 55)  = 2.85, *p* = .07. The multiple comparisons applying Bonferroni adjusted alpha levels of .0167 revealed that the slight tendency resulted from a marginal difference between the German and the Polish participants: The former (862 ms) pressed the button faster than the latter (979 ms). The button press latency was shorter in the go/color than in the go/position condition, *F*(1, 55)  = 118.76, *p*<.01. No interaction was found between the speaker groups and the attributes, *F*(2, 55)  = 0.78, *p* = .47.

**Table 1:** Mean Reaction Times (RT) in ms and Error Rates in Percentage (Standard Deviations in Parentheses).

	Condition			
	Color		Position	
Group	RT	Error	RT	Error
		Go		
Control	740 (121)	2.69 (2.57)	867 (144)	4.03 (3.23)
German	796 (153)	2.94 (2.21)	928 (144)	5.28 (3.50)
Chinese	848 (165)	2.80 (2.66)	1003 (152)	6.09 (6.16)
Polish	920 (186)	4.28 (5.04)	1038 (159)	6.28 (3.53)
		Nogo		
Control		1.25 (2.98)		8.25 (7.44)
German		0.63 (1.79)		6.75 (4.60)
Chinese		0.13 (0.57)		5.79 (6.72)
Polish		0.92 (1.71)		6.18 (6.89)

The error rates were comparable between the three speaker groups, *F*(2, 55)  = 0.51, *p* = .60. Participants made slightly more errors in the go than in the nogo conditions, *F*(1, 55)  = 3.65, *p* = .06. The error rates differed significantly between the two attributes, *F*(1, 55)  = 54.10, *p*<.01, with more errors in the position than in the color conditions. The interaction between execution decisions (go, nogo) and attributes (color, position) was significant, *F*(1, 55)  = 12.93, p<.01. The follow-up MANOVA yielded a significant effect of execution decisions, Pillai's trace  = .24, *F*(2, 37)  = 5.73, *p* = .01 and the univariate tests revealed that the error rate of color trials was greater in the go than nogo condition, *F*(1, 38)  = 6.01, *p* = .02, whereas the slight difference of position trials errors between the go and nogo conditions was not significant, *F*(1, 38)  = 3.26, *p*  = .08. None of the remaining interactions were significant (*F*s <1).

#### EEG data

**Figure 3:**
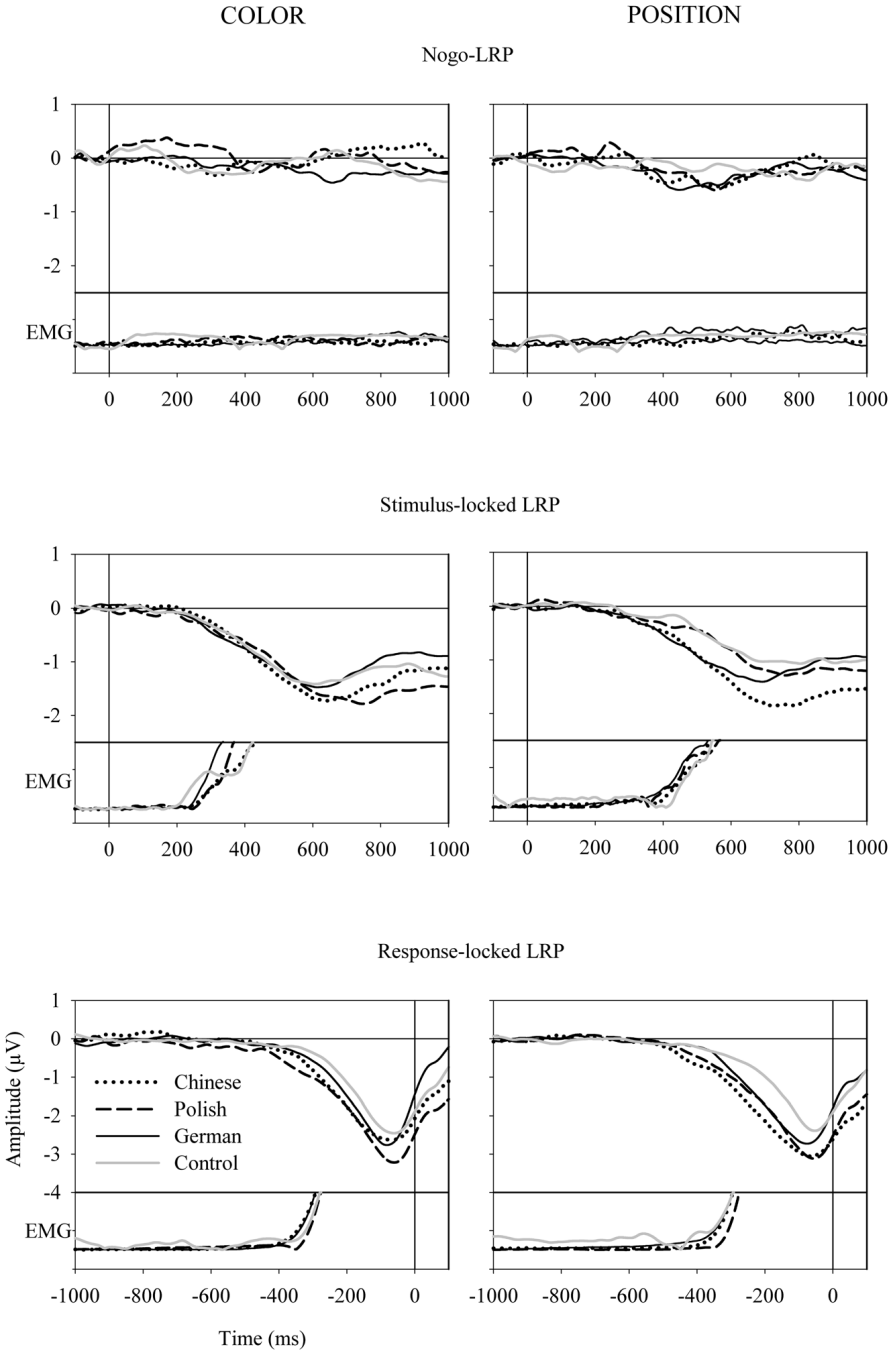
Mean lateralized readiness potentials (LRP) in the color (left charts) and the position (right charts) conditions. The three panels from top to bottom are results of the nogo-LRP, the stimulus-locked go-LRP, and the response-locked go-LRP, respectively. In the bottom of each chart the electromyogram is shown for the hand that executed the response or that would have executed the reponse which was inhibited in nogo trials. The four groups of participants were separately designated in each chart: the dotted black line for the Chinese speaker group, the solid black line for the German speaker group, the dashed black line for the Polish speaker group, and the solid gray line for the control group.

Figure 3 shows the lateralized readiness potentials in the four experimental conditions. No LRP could be confirmed in the nogo/color condition in any of the three speaker groups. In the nogo/position condition all the three speaker groups generated a negative-going LRP that persevered for more than five consecutive time windows of 20 ms.

**Figure 4:**
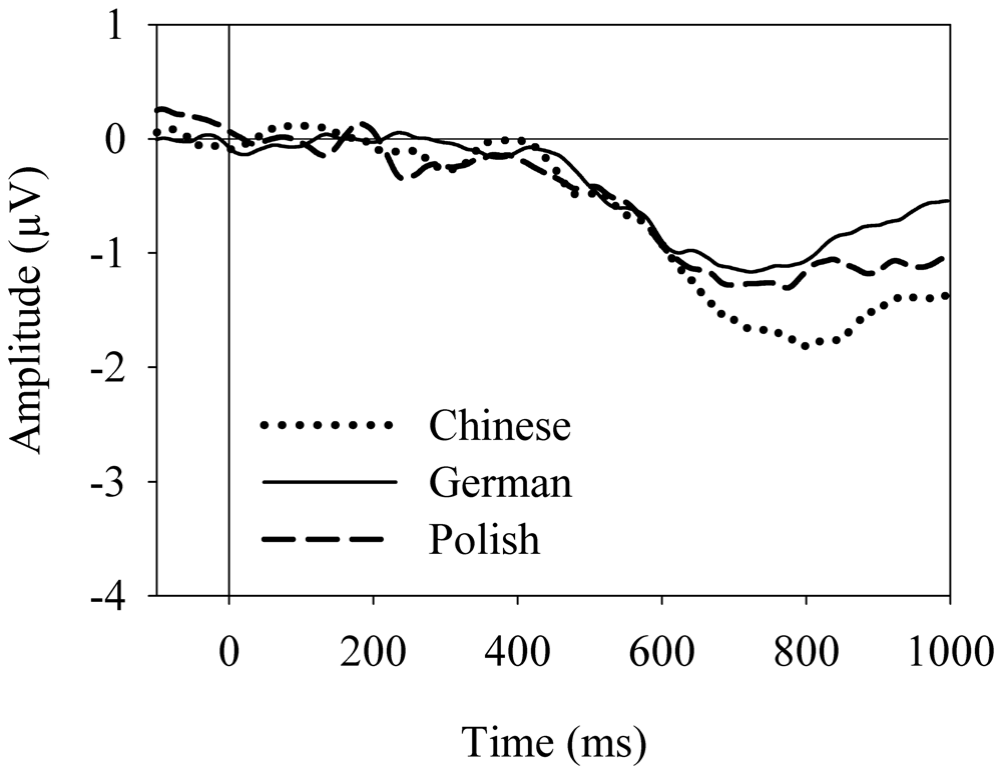
Difference waves between go- and nogo-LRP in position conditions. The difference waves were derived for the three speaker groups, respectively, by subtracting the LRP elicited in the nogo/position condition from the LRP elicited in the go/position condition: the dotted line for the Chinese speaker group, the solid line for the German speaker group, and the dashed line for the Polish speaker group.

In the analyses of the LRP onset latencies we conducted three comparisons by virtue of the lack of an LRP in the nogo/color condition. First, we compared the S-LRP-intervals in both position conditions (nogo/position, go/position). The intervals differed neither between speaker groups or the execution decisions as main effects, nor as interaction between these factors (*F*s <1). Second, we compared the S-LRP-intervals in both go conditions (go/color, go/position). The intervals did not differ between the speaker groups, *F*(2, 55)  = 0.62, *p* = .54. The difference between the attributes reached significance, *F*(1, 55)  = 6.89, *p*<.01, with the S-LRP-interval in the go/color condition (*M* = 341 ms, *SD*  = 22 ms) being shorter than in the go/position condition (*M* = 448 ms, *SD*  = 58 ms). The interaction between the speaker groups and the attributes was nonsignificant, *F*(2, 55)  = 0.95, *p* = .39. Third, we compared the LRP-R-intervals in both go conditions (go/color, go/position). No main effect of the speaker groups and the attributes or the interaction was significant (*F*s <1). Finally, when nogo decision latencies were compared between the speaker groups there was no significant difference, *F*(2, 55)  = 0.87, *p* = .43. The difference waves for estimating nogo decision latencies are presented in Figure 4.

### Discussion

The results of brain potentials revealed contradictory patterns in the two nogo conditions. In all three speaker groups an LRP emerged in the nogo/position trials, whereas no significant nogo-LRP emerged in the nogo/color trials. In the nogo/position condition the three speaker groups generated comparable LRPs in that the nogo-LRP started and ended roughly at the same time, as indicated by similar nogo-LRP onsets and nogo decision latencies, respectively. The consistent results across the speaker groups in either of the two nogo conditions might be presumed to support the notion of a consistent ordering of encoding proposition and mood of polar interrogatives in Chinese, German, and Polish alike.

However, the assumption of a unitary encoding order across languages seems difficult, if not impossible, to reconcile with contradictory findings that suggest different encoding orders, based on the nogo/color condition (e.g. proposition after mood) and on the nogo/position condition (e.g. proposition before mood). The LRP absence in the nogo/color condition indicates that no response hand was chosen due to an early nogo decision. The presence of a nogo-LRP in the nogo/position condition, on the contrary, indicates that the speakers had made the nogo decision only after choosing a response hand and preparing the hand movement. The onset latency of an LRP (i.e. the S-LRP-interval) reflects the latency of the hand choice. The comparison of the LRP onset latencies indicates that the S-LRP-interval was shorter in the go/color than in the go/position condition. This result implies that the hand choice latency was also shorter in the nogo/color than in the nogo/position condition.

According to the temporal relationship between the hand choice and the nogo decision (derived above from the LRP presence and absence), it can be inferred that the nogo decision was made earlier in the nogo/color than in the nogo/position condition. Had the nogo decision, as desired, been connected with the encoding of the interrogative mood in the experimental design, the deduction implies that the speakers processed the interrogative mood earlier when encoding a polar interrogative pertaining to color than when encoding an analogous question pertaining to position. This conclusion, however, has no solid theoretical underpinning. It seems that there is no theory of language production that would account for such a conclusion.

Thus, it is more feasible that the observed contrast in the nogo/color and the nogo/position trials originated from methodological reasons. The experimental manipulation may have worked out only in one of the nogo conditions. In the other case the motor processes of button pressing (the hand choice and the nogo decision) may have failed to connect with any linguistic encoding processes of speaking. The presence or absence of an LRP would then reflect the temporal relationship of the motor processes without linguistic connections. In this case participants would have carried out the motor task exclusively on the basis of perceptual information: The hand choice (left, right) depended on the perception of the deviant attribute (color, position) and the executive decision (go, nogo) on the perception of attribute determinability (determinable, indeterminable). If attribute perception required less time than the (in)determinability perception, a nogo LRP may have emerged by virtue of the earlier occurrence of the hand choice than the nogo decision. If the perceptual difficulty was higher for the attribute identification than for its (in)determinability, no nogo-LRP would develop. In order to clarify this issue, a control experiment was conducted.

## Control Experiment

The objective of the control experiment was to clarify the contradictory results in the nogo/color and nogo/position conditions of the main experiment by using the same motor tasks as before but without requiring language production. The data of the control group should determine whether the motor processes of the button pressing task were connected with the linguistic processes of the speaking task. Note that a conclusion might be drawn only if the result of a nogo condition in the control experiment contrasts with its counterpart in the main experiment. That is, when an LRP appeared in the nogo/color condition of the control experiment, its absence in the main experiment must have been related to the speaking task. By the same token, when the control group generated no LRP in the nogo/position condition, the linguistic processes must have led to its presence of the speaker groups. Therefore, for direct comparisons the data analyses incorporated both the control and the three speaker groups.

### Method

#### Participants

Twenty-two German students (15 female, aged 19 to 32) took part in the control experiment. Two participants (one female) were discarded from the analyses because they made errors in more than 25% of the trials in the nogo/position condition. All participants were right-handed and had normal or corrected-to-normal visual acuity. They were either paid or received credits required for the psychology curriculum.

#### Procedure

The same six lists of identical materials created for the main experiment were also used in the control experiment. The control experiment differed from the main experiment only for a few procedural modifications and in the instructions. First, participants were asked to perform the button pressing task, while speaking was not mentioned. Second, participants did not name objects at the beginning of a session. The first four practice blocks were also omitted due to the exclusion of the speaking task. Third, the speech bubble accompanying the signaling caricature was removed to avoid any association with speech. Few participants asked about the caricature during the session and no participant attempted to figure out the motive behind the caricature. The rest of the procedure was identical with the main experiment.

### Results

#### Behavioral data

Table 1 shows the means and standard deviations of reaction times and error rates. The reaction times differed between the four groups, *F*(3, 74)  = 5.43, *p*<.01. The multiple comparisons applying Bonferroni adjusted alpha levels of .0167 revealed that the control group was considerably faster than the Chinese and the Polish groups, whereas the difference between the control and the German speaker groups was not significant. The fast response of the German groups may be due to the fact that most of the German participants regularly took part in psychological experiments and only a few participants of the Chinese and Polish groups had such experiences. Overall, reaction time was shorter in the go/color than in the go/position condition, *F*(1, 74)  = 124.04, *p*<.01. No interaction was found between the four groups and the attributes, *F*(3, 74)  = 0.44, *p* = .73.

The error rates were similar not merely for the four groups, *F*(3, 74)  = 0.31, *p* = .60, but also between the execution decisions (go, nogo), *F*(1, 74)  = 0.31, *p* = .82. However, error rates differed significantly between the two attributes (color, position), *F*(1, 74)  = 72.21, *p*<.01, with more errors in the position than in the color conditions. The interaction between the execution decisions and the attributes was significant, *F*(1, 74)  = 23.51, *p*<.01. The participants made more errors in the nogo/position than in the nogo/color condition, whereas the error rates were similar in the go/position and the go/color conditions. None of the remaining interactions were significant (*F*s <1).

#### EEG data

Figure 3 shows the lateralized readiness potentials under the four experimental conditions. The control groups generated no LRP in either of the two nogo conditions.

In the analyses of the LRP onset latencies we compared the S-LRP- and the LRP-R-intervals of the four groups in the go conditions (go/color, go/position). First, the S-LRP-intervals did not differ between the four groups, *F*(3, 74)  = 0.83, *p* = .48. The difference between the attributes reached the significance level, *F*(1, 74)  = 13.09, *p*<.01, with the S-LRP-interval in the go/color condition (*M* = 347 ms, *SD*  = 22 ms) being shorter than in the go/position condition (*M* = 467 ms, *SD*  = 60 ms). The interaction between the four groups and the attributes was nonsignificant, *F*(3, 74)  = 0.86, *p* = .59. Second, the LRP-R-intervals differed between the four groups, *F*(3, 74)  = 3.06, *p* = .03. The multiple comparisons revealed that this interval was shorter in the control group (*M* = 112 ms, *SD*  = 14 ms) than in the Chinese (*M* = 191 ms, *SD*  = 19 ms), *t*(37)  = 3.02, *p*<.01, the German (*M* = 160 ms, *SD*  = 10 ms), *t*(38)  = 1.93, *p* = .06, and the Polish speaker group (*M* = 169 ms, *SD*  = 7 ms), *t*(37)  = 2.11, *p* = .04. No significant difference of the LRP-R-intervals was found between the attributes, *F*(1, 74)  = 0.18, *p* = .67. The interaction between the four groups and the attributes was nonsignificant either, *F*(3, 74)  = 1.27, *p* = .29.

In addition, a one-way ANOVA was conducted to compare the average amplitudes of stimulus-locked go-LRP between the four groups. Roughly estimating, the nogo-LRP of the three speaking groups was present from 300 ms to 700 ms after the probe onset. The comparison of the average amplitudes in this period of 400 ms yielded no significant difference, *F*(3, 74)  = 1.81, *p* = .15.

### Discussion

The control experiment aimed to elucidate whether and in which condition of the main experiment the motor processes of the button press had been connected with the linguistic processes of the speaking task. An LRP was elicited from the control group neither in the nogo/color nor in the nogo/position condition. In the main experiment no LRP was present in the nogo/color condition either. The absence of a nogo-LRP in the nogo/color conditions of both control and main experiment might indicate a failure to form a connection between the sensorimotor button pressing task and the linguistic processes required for speaking. In the nogo/position condition, on the contrary, the absence of a nogo-LRP in the control group contrasted with its presence in all speaker groups of the main experiment. The emergence of a nogo-LRP in the main experiment may therefore be attributed to the linguistic processes of the utterance production.

The conclusion is tenable, nonetheless, only with the premise that there is no other systematic distinction in the position conditions between the main and control experiments. One of the possible confounding variables pertains to the difference of task complexity: The speaking groups had to carry out both button pressing and speaking tasks, whereas the control group needed to perform only the former. Accordingly, if the amplitude of the go-LRP had been generally greater in the speaking than in the control group, the absence of nogo-LRP in the control group could be ascribed to the globally reduced LRP in the control group due to diminished task complexity for this group. However, no significant difference of go-LRP amplitudes was found between the two experiments. Therefore, the empirical data do not lend support to covariance between task complexity and LRP amplitude: The differential LRP effects between the control and speaking groups were confined to the nogo conditions and did not covary with differences in the go conditions.

In addition to the lack of empirical support, a further problem of the task complexity account is the presumably selective impact on perceptual processes. According to this explanation, the more complex task in the main experiment might have led speakers to allocate less resources to those perceptual processes leading to the execution decision (go vs. nogo). A reduction of resource allocation would postpone the withdrawal decision in the nogo condition, giving rise to a nogo-LRP. This explanation seems to imply that the process of execution decision be more difficult than that of hand choice and, consequently, would be specifically affected by reduced resources deployment. However, if execution decision is inherently more difficult than hand choice, it should always demand more processing time and evoke a nogo-LRP in any case. However, no nogo-LRP was found in the control experiment, which is at odds with the presumption above.

## General Discussion

In the present investigation we examined the processing of the sentence mood of a polar interrogative at the semantic-syntactic interface in Chinese, German, and Polish. In the experimental design we endeavored to connect the motor processes of button pressing, which elicited the lateralized readiness potentials at the scalp, with the linguistic processes of semantically structuring an utterance. The collected data indicated that the three speaker groups encoded the interrogative mood at a comparable time point. In the following we first scrutinize the validity of the experimental paradigm. We will take the position that the findings remain viable within methodological limitations of the experiments. Then we discuss the theoretical account of the findings, which will lead to the conclusion that the results suggest a consistent order of encoding the interrogative and the proposition during generation of the semantic structure of a polar interrogative in the three languages.

The result patterns in the nogo/color and nogo/position conditions of the main and control experiments are distinct from each other in terms of the presence of an LRP: No LRP emerged in the nogo/color condition of both experiments; in contrast, whereas an LRP was present in the nogo/position condition of the main experiment, it was absent in the same condition of the control experiment. The analyses of the stimulus- and response-locked LRP onset latencies shed more light on the distinction between the color and position conditions. The comparison of these intervals revealed that all four groups yielded similar S-LRP-intervals, whereas the LRP-R-intervals differed primarily between the control and the speaker groups. That is, the effect of speaking was manifest in cognitive processes after the LRP onset. Furthermore, the S-LRP-interval was substantially shorter in the go/color than in the go/position condition, whereas no significant difference of the LRP-R-intervals was found between the two attributes. These findings imply that the functional locus of the discrepancy between color and position is different from that of the speaking effect.

It is likely that in the nogo/color condition of the main experiment participants had carried out the tasks of button pressing and speaking separately because omitting speaking in the control experiment did not alter sensorimotor performance nor ERP-indicators of processing. The results of the brain potentials thus reflected the temporal relationship of the two motor processes, the hand choice and the nogo decision, whose temporal relationship was contingent upon stimulus perception and unrelated to any linguistic processing.

The analyses of the behavioral data indicate that reaction times were shorter in the go/color than in the go/position condition and error rates were noticeably lower in the nogo/color than in the nogo/position condition. Both results point to the easier perception of the more conspicuous color contrast between the target and the probe pictures. As a consequence, the speakers may have had enough time and become inclined to temporally separate the two tasks by accomplishing button pressing and speaking in succession. In this regard, the color conditions did not seem conducive for the purpose of the present investigation.

The contrasting findings in the nogo/position condition of the main and the control experiment (presence vs. absence of a nogo-LRP), on the other hand, hint at the effectiveness of the experimental manipulation. That is, the hand choice was connected with the encoding of the propositional content and the nogo decision with the encoding of the interrogative mood in this condition. The nogo-LRP emergence of the three speaker groups, which arose from the hand choice followed by the nogo decision, therefore indicates that the interrogative mood was processed earlier than the propositional content. As a result, when semantically structuring a polar interrogative, the native speakers of Chinese, German, and Polish all encoded the two sentential parts in the implicated order.

However, the conclusion made above about the encoding sequence, namely proposition before interrogative mood, is no more tenable in light of the analyses of the LRP onset latencies. The analyses yielded that a) the S-LRP-intervals were comparable in the go/position conditions of the control and the main experiments and b) the S-LRP-intervals of the speaker groups did not differ between the go/position and the nogo/position condition. The two results suggest that the LRP onset latencies were similar in the go/position condition of the control experiment and in the nogo/position condition of the main experiment. That is, according to the S-LRP onsets, the hand choice required the same amount of time whether or not the speaking task was involved. Following the reasoning that no certain conclusion should be drawn from the absence of a difference between the control and the speaker groups, we can no longer ascertain that the *hand choice* was connected with the encoding of the propositional content in the nogo/position condition of the main experiment.

We argue, nevertheless, for a consistent order of encoding of the two sentential parts in the three languages, albeit the specific order can not be ascertained on the basis of the collected data. The argument is based on the analyses of the processing latencies of the interrogative mood. First, the *nogo decision* was effectively connected with the encoding of the interrogative mood: If the hand choice had not been connected with any linguistic process in the nogo/position condition of the main experiment, its processing latency should have been the same in the corresponding condition of the control experiment, which was not the case. Furthermore, the presence of nogo-LRPs in the speaker groups revealed the sequence of selecting the response hand prior to making nogo decisions, whereas the absence of a nogo-LRP in the control group implies completion of the nogo decision prior to hand selection. Therefore, given that the hand selection latency was comparable in the nogo/position condition of the main and the control experiments, the presence of a nogo-LRP in the speaker groups indicates that it had been the nogo decision that was postponed by speaking. The effect was related to the linguistic processing of utterance production, plausibly due to the effective manipulation in the experimental design, namely the connection of the nogo decision with the encoding of the interrogative mood.

Second, the analyses of the nogo decision latencies yielded no difference between the three speaker groups in the nogo/position condition. Now that the nogo decision can be connected to processing of the interrogative mood and given that its latencies in the three speaker groups are similar, it can be inferred that the interrogative mood was encoded at roughly the same time in Chinese, German, and Polish. The inference may be applied to assess two hypothetical orders of encoding the proposition and the interrogative mood during the semantic structuring of a polar interrogative. According to the incremental hypothesis the two sentential parts are semantically processed in the sequence of their grammatical formulation. That is, the interrogative mood is encoded in German and Polish before the proposition, but thereafter in Chinese. However, given the concurrent encoding of the interrogative mood in the three languages, the incremental hypothesis implies that the generation of the semantic structure of a polar interrogative is implemented in German and Polish (commenced by processing the proposition before the interrogative mood) earlier than in Chinese (commenced by encoding the interrogative mood before the proposition). The implication must be reasonably challenged in that, upon an identical probe stimulus, the German and the Polish speakers did not initiate the utterance sooner than the Chinese speakers.

According to the consistency hypothesis, on the contrary, the order of encoding the two sentential parts is the same in the three languages. Whether the interrogative mood is consistently encoded before or after the proposition, the semantic structuring of a polar interrogative starts concurrently in Chinese, German, and Polish. The findings of the present study hence corroborate the consistency rather than the incrementality hypothesis.

A methodological concern bears on the processing level of utterance production measured in the main experiment. An LRP was elicited from the speakers in the nogo/position condition, which hinted at an effective connection of the nogo decision with the interrogative mood processing. On the other hand, the experimental design did not impose constraints on the processing level at which the connection was established. Therefore, instead of the semantic structuring, it might be the grammatical formulation, the phonological specification, or the motor programming for articulation during which the encoding of the interrogative mood was connected with the nogo decision. However, the incremental tenet of utterance production would rule out those possible loci of the connection. Whereas incrementality may be limited from the semantic processing to the syntactic framing [47], the mapping of linguistic forms and articulatory gestures is rather straightforward. In order to construct the sentence frame of a Polish polar interrogative, for instance, the interrogative particle has to be syntactically ordered before the proposition. In the ensuing processes its phonological shape, the phonetic gesture, and the corresponding motor program all will be produced before the counterparts of the proposition. In Chinese the form encoding and the articulatory programming of the two sentential parts are implemented in the converse order. As a consequence, if the encoding of the interrogative mood had been connected with the nogo decision at any of those levels, the nogo decision would have been made at disparate time points in Chinese and Polish. Now that the derived anticipation contradicts with our observations, it seems unfeasible that the connection was established beyond syntactic word ordering.

The experimental results of the present study suggest that, with respect to processing order, the encoding of the interrogative mood is consistent across languages when the semantic structure is generated for a polar interrogative in a specific form. On the basis of the findings, we propose that the sentence mood in other forms of interrogatives is semantically encoded in the same way, irrespective of its syntactic encoding at diverse points in the surface structures. Content interrogatives, for example, make use of interrogative words to request a missing piece of information. In some languages such as Chinese and Japanese the word order of a content interrogative is identical with the declarative counterpart. The interrogative word replaces the missing constituent in situ. In other languages such as English and German the interrogative word of a content question usually appears in the initial position of the sentence. Sometimes the interrogative word is uttered in situ for emphasis (e.g. *He did what*?). Similarly, languages mark polar interrogatives in various ways – among others word order, interrogative particle, and intonation. Each of those linguistic devices sets the marking of the interrogative mood at a specific point of the surface structure in a given language. Moreover, languages often combine devices to express the same polar interrogative.

The variety of syntactic markers of interrogatives indicates that the interrogative mood is syntactically specified at distinctive points in the surface structures. It is likely that, during the semantic structuring of interrogatives, the interrogative mood is not encoded according to its grammatical processing. Instead, in light of the present findings, the semantic encoding of the interrogative mood may not differ between languages, whether the question is a polar or content interrogative, in one or another form.

In conclusion, given a proposition, languages differ in the time point at which the sentence mood is marked in the surface structure of interrogatives. The present study endeavored to explore whether the encoding of the interrogative mood is differential and corresponds to the variations of its grammatical specification when a polar interrogative is semantically structured in different languages. At variance with this idea, the present electrophysiological results did not substantiate a temporal disparity of the encoding and, instead, support a consistent temporal ordering of interrogative mood and proposition. The findings drop a hint at theoretical elaboration of how the sentence mood is represented and processed at the semantic-syntactic interface of utterance production. Such theoretical grounding will facilitate experimental designs in future research on the processing of utterance modalities.
